# KRSR and RGD Adsorption on TiO_2_ and Influence of Ion Concentration: A Molecular Dynamics Study

**DOI:** 10.3390/biom16020336

**Published:** 2026-02-23

**Authors:** Tamás Tarjányi, Csaba Ákos Rosztóczy, Tibor Szabó

**Affiliations:** Department of Medical Physics and Informatics, University of Szeged, Korányi Fasor 9, H-6720 Szeged, Hungary; rosztoczy.csaba@gmail.com (C.Á.R.); tiberatosz@gmail.com (T.S.)

**Keywords:** molecular dynamics, peptide, TiO_2_, adsorption, KRSR, RGD

## Abstract

Bioactive peptide coatings modulate cell–implant interactions on TiO_2_ surfaces; however, most molecular-level studies of peptide adsorption are performed under low or fixed ionic conditions. Physiological environments exhibit non-negligible and variable electrolyte concentrations, so understanding ionic strength effects is crucial for designing effective peptide-functionalized titanium implants. An amorphous TiO_2_ surface was generated from a crystalline rutile precursor and simulated in explicit water using classical molecular dynamics at nine NaCl concentrations. For each condition, seven independent simulations with different initial peptide placements/orientations were performed. Peptide backbone RMSD, minimum peptide–surface distance, and adsorption time ratio were analysed as functions of NaCl concentration. For both peptides, backbone RMSD remained stable and showed no statistically significant correlation with NaCl concentration. KRSR exhibited a significant increase in minimum distance with increasing NaCl concentration and a significant decrease in adsorption time ratio, indicating reduced persistence of close surface contact at higher salt levels. In contrast, RGD showed no significant dependence of either minimum distance or adsorption time ratio within the tested range. Within the limits of the applied force-field MD framework and the investigated NaCl range, KRSR adsorption on TiO_2_ is more sensitive to ionic strength than RGD, consistent with the stronger electrostatic contribution for the net-positively charged KRSR motif.

## 1. Introduction

Nowadays titanium (Ti) and its alloys are commonly used for load-bearing implants both in medicine and dentistry due to its remarkable mechanical behaviour, corrosion resistance, and outstanding biocompatibility [1,2]. A key factor underlying the clinical success of Ti-based implants is their ability to achieve stable osseointegration, defined as the direct structural and functional connection between living bone tissue and the implant surface [3]. This property makes titanium an ideal material for orthopaedic joint replacements and dental implants.

Under physiological conditions, Ti surfaces are spontaneously covered by a thin, stable oxide layer, predominantly composed of titanium dioxide (TiO_2_) [4]. Consequently, this oxide layer interacts mainly with the biological environment rather than the metallic titanium itself. During the early stages of implantation, a complex biolayer forms rapidly on the TiO_2_ surface as biomolecules from blood plasma and interstitial fluids adsorb onto the implant [5]. This adsorbed molecular layer plays a decisive role in subsequent cellular responses, including osteoblast adhesion, proliferation, and differentiation, resulting in the quality and long-term stability of osseointegration.

The biolayer consists of a wide range of biomolecules, including proteins, peptides, and other extracellular matrix components [6,7,8]. Among these, short bioactive peptide motifs have attracted considerable attention due to their ability to modulate cell–surface interactions in a controlled manner. In particular, the RGD (arginine–glycine–aspartic acid) and KRSR (lysine–arginine–serine–arginine) peptide sequences are well known for promoting cell adhesion and osteogenic activity on TiO_2_ surfaces [9,10]. The RGD motif is a three amino acid sequence which is present in several extracellular matrix proteins and mediates cell adhesion via integrin receptors, playing a central role in bone–implant interactions. The KRSR tetrapeptide selectively binds to bone-associated proteoglycans and has been shown to enhance osteoblast adhesion while limiting fibroblast attachment, making it particularly attractive for orthopaedic and dental applications.

In recent years, molecular dynamics (MD) simulations, often complemented by ab initio and density functional theory (DFT) calculations, have become powerful tools for investigating the molecular mechanisms underlying peptide adsorption on TiO_2_ surfaces [11,12]. Numerous computational studies have explored the interactions of RGD, KRSR, and related peptides with different TiO_2_ polymorphs, most commonly rutile and anatase, providing valuable insights into adsorption geometries, binding energies, and dominant interaction motifs [13,14]. A comprehensive overview of these theoretical advances is provided in the review by Blazhynska et al., summarizing recent progress in MD and quantum chemical studies of TiO_2_ nanomaterials and their biointeractions [15].

Early molecular mechanics and MD studies demonstrated that the physicochemical properties of hydrated TiO_2_ surfaces like surface hydroxylation, charge regulation, and interfacial water structure play an important role in biomolecule adsorption [16,17]. Several recent MD studies have subsequently addressed biomolecule adsorption on TiO_2_ surfaces at atomic resolution. Brandt and Lyubartsev performed MD simulations of amino acid side chain analogues and a titanium-binding peptide on a hydrated TiO_2_ (100) surface, revealing how local surface features and solvation effects govern specific adsorption motifs [18]. Agrelli et al. applied an in silico approach to design and evaluate peptides for coating TiO_2_ implants with the aim of improving osseointegration, highlighting the potential of computational screening for peptide functionalization strategies [19]. Rahmani and Lyubartsev systematically explored the adsorption of 360 six-residue titanium-binding peptide permutations on hydroxylated anatase TiO_2_, demonstrating that peptide sequence order can strongly affect binding strength and conformational behaviour at the interface [20].

Despite these advances, the influence of ionic strength and electrolyte composition on peptide-TiO_2_ interactions has received comparatively little attention. This is a notable limitation, as physiological environments are characterized by non-negligible and variable salt concentrations, which can significantly modulate electrostatic screening, peptide conformation, surface charge states, and adsorption stability. Understanding how ionic conditions affect peptide binding is therefore essential for bridging the gap between idealized simulation setups and realistic biological environments.

In this study, we systematically investigated the adsorption behaviour of RGD and KRSR peptides on an amorphous TiO_2_ surface, which was generated from an initially crystalline rutile structure in order to capture the structural disorder characteristic of experimentally relevant implant surfaces. MD simulations were performed at nine different NaCl concentrations, covering a range of physiologically relevant ionic strengths. For each ionic condition, seven independent initial peptide configurations were generated to ensure robust statistical sampling. By analysing adsorption kinetics, peptide–surface contact patterns, and the effect of ionic strength on binding stability, this work provides a detailed molecular-level understanding of how electrolyte conditions modulate peptide–implant surface interactions. These insights contribute to a better mechanistic understanding of peptide adsorption on TiO_2_ surfaces and may support the rational design of biofunctionalized titanium implants with improved osseointegration and clinical performance.

## 2. Materials and Methods

### 2.1. System Preparation

The MD simulation setup and parameterization procedure followed protocols established in our previous studies and the related literature [21,22]. All simulations were performed with GROMACS (version 2023.3) [23,24,25]. The RGD and KRSR peptide structures were built using the builder module Gabedit (version 2.5.0) [26]. Biomolecular interactions were described using the CHARMM36 force field, which was applied consistently to peptides, ions, and solvent molecules [27]. Water was modelled using the TIP3p model [28].

**Figure 1:**
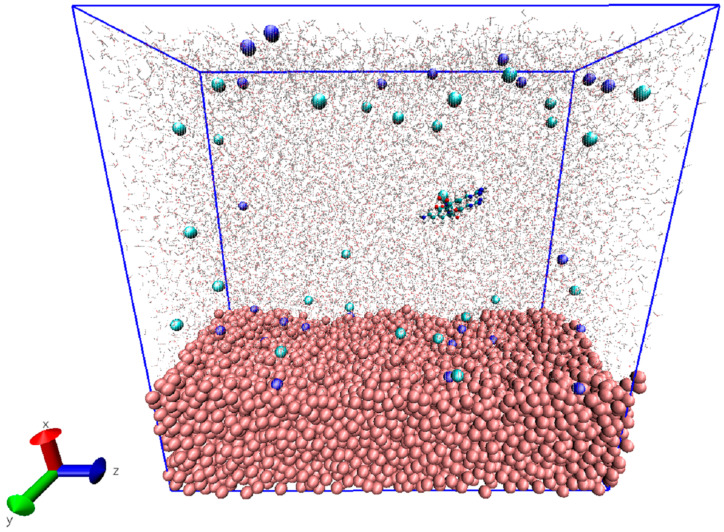
Overview of the molecular dynamics simulation box used in the study. The amorphous TiO_2_ slab is shown at the bottom in pink VDW representation. Sodium and chloride ions are shown cyan and dark blue; peptide is shown in standard CPK representation. Water molecules are displayed transparently to visualize the spatial arrangement of all components within the simulation box.

The crystalline rutile TiO_2_ unit cell was obtained from the American Mineralogist Crystal Structure Database and subsequently used as the starting point for generating the amorphous TiO_2_ surface model [29]. Structural amorphization was achieved using a melt–quench–relaxation procedure, yielding a disordered TiO_2_ surface representative of experimentally relevant implant surfaces, following the protocol of our previous paper [21]. The resulting amorphous TiO_2_ slab was 2.1 × 4.90 × 10.70 nm^3^ containing 3475 Ti and 6950 O atoms; see Figure 1. Force field parameters for TiO_2_ were adopted from the work of Luan et al. [30], which has been successfully applied in our previous MD studies of biomolecule-TiO_2_ interfaces [21,22]. This parametrization reproduces hydration structure, surface electrostatics, and adsorption energetics of TiO_2_ surfaces in aqueous environments. The Lennard-Jones and partial charge parameters used for Ti and O atoms were as follows: $\varepsilon_{O\text{–}O}=1.297$ kJ/mol, $\varepsilon_{Ti\text{–}O}=1.774$ kJ/mol, $\varepsilon_{Ti\text{–}Ti}=2.427$ kJ/mol, $\sigma_{O\text{–}O}=0.324$ nm, $\sigma_{Ti\text{–}O}=0.272$ nm, $\sigma_{Ti\text{–}Ti}=0.22$ nm, $q_{O}=\text{–}1.098$ e, $q_{Ti}=+2.196$ e.

The dimension in the x direction of the simulation box was extended, and the final box size was 10.11 nm × 4.90 nm × 10.70 nm. Each simulation system was solvated in a rectangular box using explicit TIP3P water molecules. To investigate the influence of electrolyte conditions, nine different NaCl concentrations were prepared by randomly replacing water molecules with Na^+^ and Cl^−^ ions. The resulting salt concentration ranged from 1 mM to 200 mM—see Table 1—covering dilute to physiologically relevant extracellular ionic strength. Overall charge neutrality of each system was maintained. The GROMACS raw files can be downloaded found in the Supplementary Materials including the input files, scripts and analysis outputs.

### 2.2. Energy Minimization and Equilibration

Prior to the equilibration step, all systems were subjected to energy minimization using the steepest descent algorithm in order to remove steric contacts and high-energy atomic overlaps introduced during system construction. The steps were set to 50,000, or until the maximum force dropped below 1000 kJ/mol/nm.

After this, an equilibration procedure was carried out in two stages using 2 fs integration time step and the leap-frog integrator:

- NVT equilibration (200 ps): The system temperature was set to 310 K using the velocity-rescale (V-rescale) thermostat [31], with separate coupling groups for peptide and non-peptide components and time constant of 0.1 ps.
- NPT equilibration (250 ps): Pressure was controlled at 1 bar using the Parinello–Rahman barostat [32] with a time constant of 0.2 ps, while temperature control was maintained with the V-rescale thermostat.

During both equilibration phases, periodic boundary conditions (PBC) were applied in all three spatial dimensions. To prevent premature surface binding and allow proper solvent relaxation, the heavy atoms of the peptides were restrained with a harmonic position restraint of 1000 kJ/mol/nm^2^.

### 2.3. Production Simulations

For each NaCl concentration, seven independent production simulations were performed with different initial orientations and positions relative to the TiO_2_ surface. The peptides were initially placed at random lateral positions and distances from the surface to ensure unbiased sampling of adsorption pathways. Each production run was carried out for 50 ns in the NPT ensemble without the previously mentioned restrictions on the peptides. Temperature was maintained at 310 K using the Nosé-Hoover thermostat [33,34], ensuring proper canonical ensemble sampling, while pressure was controlled at 1 bar using the C-rescale barostat [31]. PBC was applied in all three direction for the production simulations. Long-range electrostatic interactions were treated using the particle mesh Ewald (PME) method [35], and standard cutoff schemes were applied for short-range nonbonded interactions.

## 3. Results

### 3.1. Stuctural Stability of RGD and KRSR Peptides

**Figure 2:**
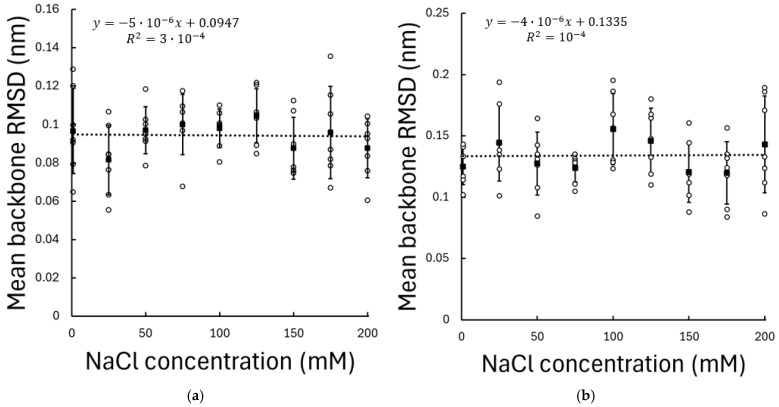
Mean backbone RMSD of (**a**) RGD and (**b**) KRSR peptides adsorbed on the amorphous TiO_2_ surface as a function of NaCl concentration. RMSD values were calculated relative to the equilibrated reference structure and averaged over the production phase of the simulations. Only trajectory frames in which the peptide was located within 1 nm of the TiO_2_ surface were included in the analysis. Data points represent the mean RMSD obtained from seven independent simulations at each ionic concentration, while error bars indicate the standard deviation. Dashed lines represent the linear regression fits to the data.

The structural stability of the peptides was evaluated by calculating the backbone root-mean-square deviation (RMSD) for both RGD and KRSR across all NaCl concentrations. For both peptides, RMSD values remained stable throughout the simulations, indicating equilibrated conformational behaviour under all investigated ionic conditions. No statistically significant correlation was observed between RMSD and NaCl concentration (RGD: p=0.889, r=−0.0179; KRSR: p=0.939, r=0.01), either for RGD or KRSR; see Figure 2. The average RMSD values exhibited only minor fluctuations across concentrations and remained within a narrow range for all seven independent replicas. To ensure that RMSD reflects peptide conformational stability rather than bulk translational diffusion, only trajectory frames in which the peptide was located within 1 nm of the TiO_2_ surface were included in the analysis.

These results indicate that variations in ionic strength do not induce substantial conformational rearrangements of the peptides and that the peptide backbones remain structurally stable over the investigated salt concentration range. Consequently, any observed differences in adsorption behaviour or surface interactions at different ionic strengths cannot be attributed to global peptide destabilization.

### 3.2. Distance to the Surface

To quantify peptide–surface proximity and adsorption behaviour, the mean minimum distance between the peptides and the TiO_2_ surface was analysed as a function of NaCl concentration from the average of each simulation.

**Figure 3:**
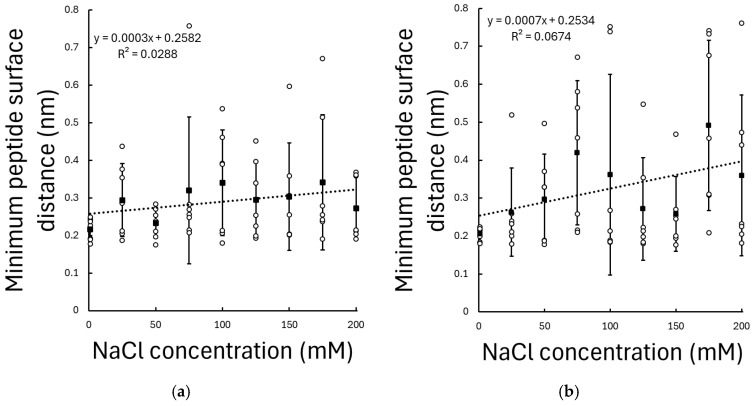
Mean minimum distance between (**a**) RGD and (**b**) KRSR peptides and the amorphous TiO_2_ surface as a function of NaCl concentration. Only trajectory frames in which the peptide was located within 1 nm of the TiO_2_ surface were included in the analysis. Values are averaged over seven independent simulations per condition; error bars indicate standard deviation. Dashed lines represent the linear regression fits to the data.

For the KRSR peptide, a statistically significant positive correlation (p=0.04, r=0.26) was observed between NaCl concentration and the mean minimum peptide–surface distance; see Figure 3. Increasing ionic strength resulted in a systematic increase in the mean minimum distance, with a slope of 0.718 pm/mM, indicating that KRSR tends to reside farther from the TiO_2_ surface at higher ionic concentrations. For example, at 100 mM the mean distance is increased by 0.072 nm, which is approximately an atomic distance.

In contrast, the RGD peptide exhibited only a weak and statistically non-significant relationship between NaCl concentration and mean minimum distance (p = 0.184, r = 0.17), with mean values remaining within a relatively narrow range across all ionic conditions; see Figure 3.

These results demonstrate a pronounced peptide-specific sensitivity to ionic strength, with the adsorption of the positively charged KRSR peptide being strongly modulated by electrolyte concentration, while the largely neutral (but polar) RGD peptide remains comparatively insensitive to changes in ionic strength.

### 3.3. Adsorption Dynamics

To directly quantify adsorption dynamics, the fraction of simulation time during which the peptides remained in close contact with the TiO_2_ surface was evaluated. Adsorption dynamics were quantified by calculating the adsorption time ratio, defined as the fraction of the simulation time during which the peptide was located within 0.3 nm of the TiO_2_ surface relative to the non-adsorbed state.

**Figure 4:**
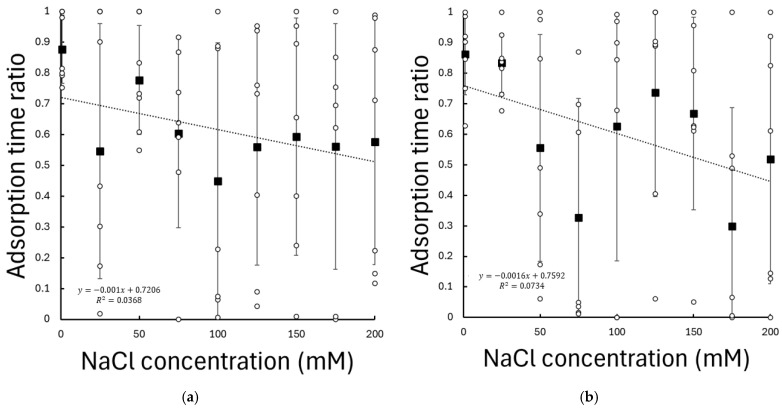
Adsorption time ratio of (**a**) RGD and (**b**) KRSR peptides on the amorphous TiO_2_ surface as a function of NaCl concentration. The adsorption time ratio was defined as the fraction of simulation time during which the peptide–surface distance was below 0.3 nm. Values represent means over seven independent simulations per condition; error bars indicate standard deviation. Dashed lines represent the linear regression fits to the data.

For the KRSR peptide, the adsorption time ratio showed a statistically significant (p=0.032, r=−0.271) dependence on NaCl concentration; see Figure 4. Increasing ionic strength resulted in a systematic reduction of the time spent in close surface contact, with a slope of −0.156%/mM, indicating that KRSR adsorption becomes progressively less persistent at higher NaCl concentrations.

In contrast, the adsorption time ratio of the RGD peptide showed no statistically significant correlation with the NaCl concentration (p=0.132, r=−0.192). RGD maintained comparable surface residence times under all investigated ionic conditions; see Figure 4.

These results demonstrate a pronounced peptide-specific sensitivity of adsorption dynamics to ionic strength, with the positively charged KRSR peptide being affected by electrolyte concentration, whereas the largely neutral RGD peptide displays similar adsorption behaviour over the same concentration range.

### 3.4. Dynamics of the Amino Acid Residues of the Peptides

To characterize the residue-specific binding conformations, the minimum distances of individual amino acid residues relative to the TiO_2_ surface were analysed throughout the MD trajectories. The resulting distance histograms revealed two well-defined maxima at approximately 0.2 nm and 0.4 nm, corresponding to distinct adsorption regimes. Gaussian functions were fitted to these peaks to define discrete residue–surface interaction states. Residues located at ~0.2 nm were assigned to state C, representing direct surface contact (denoted by C as close contact; this is a primary adsorption layer), whereas residues at ~0.4 nm were classified as state S, corresponding to solvent-separated, water-mediated interactions (this is a secondary adsorption layer and is denoted by S as solvent-separated adsorption). Residues located beyond these distance ranges were assigned to state N, representing non-adsorbed configurations. Based on these definitions, the trajectories were segmented to quantify the occurrence of specific combinatorial residue–surface conformations.

**Figure 5:**
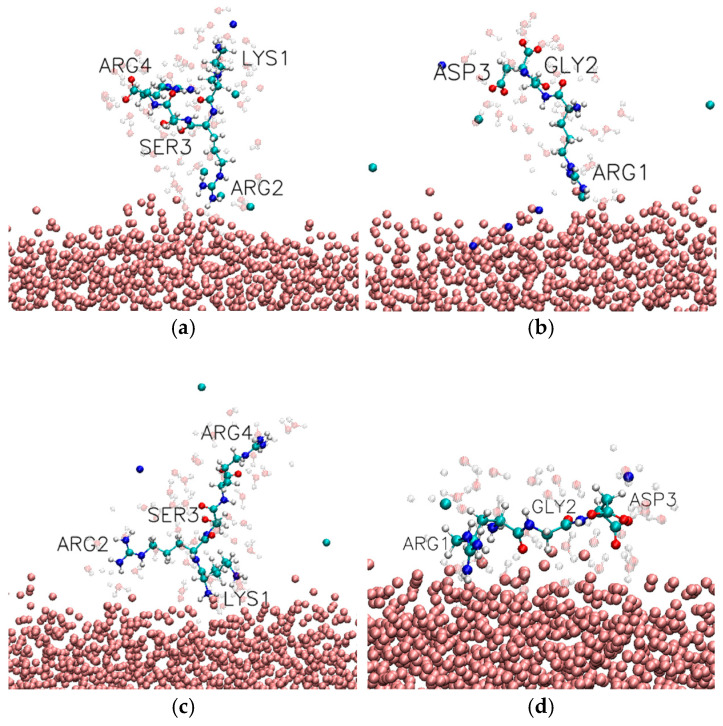
Representative MD snapshots illustrating distinct adsorption modes of short peptides on the TiO_2_ surface. (**a**,**c**) KRSR peptide conformations: in (**a**), the second arginine residue is in direct contact with the surface, while the remaining residues remain solvated; in (**c**), adsorption occurs via the N-terminal lysine. (**b**,**d**) RGD peptide conformations: in (**b**), adsorption is mediated by the N-terminal arginine; in (**d**), both arginine and aspartate residues simultaneously interact with the TiO_2_ surface. The amorphous TiO_2_ is shown in pink CPK representation, sodium and chloride ions are shown cyan and dark blue; peptide is shown in standard CPK representation, close water molecules are shown transparently.

Among the adsorbed configurations, the KRSR peptide exhibited a pronounced preference for anchoring via the N-terminal lysine residue. The most frequently observed bound conformation was C-N-N-N (10.35%), in which Lys residue is in direct contact with the TiO_2_ surface while the remaining residues remain fully solvated (see Figure 5c and Table 2). In addition to this dominant single-point attachment, cooperative binding modes were also observed. Notably, the C-C-N-N (3.82%) configuration indicates simultaneous direct contact of Lys and the adjacent Arg, suggesting transient multivalent anchoring. Furthermore, bridged conformations such as C-N-N-C (4.44%) were observed, in which both terminal residues (Lys and Arg) interact directly with the surface while the central residues remain detached, reflecting an extended peptide geometry at the interface.

**Figure 6:**
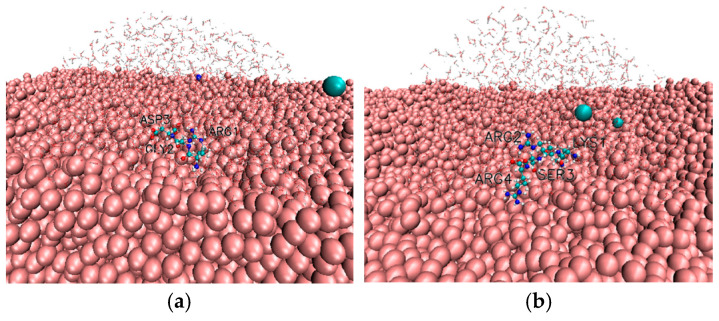
(**a**) RGD and (**b**) KRSR adsorbed on amorphous TiO_2_ (tilted view). The amorphous TiO_2_ is shown in VDW pink representation, sodium and chloride ions are shown cyan and dark blue; peptide is shown in standard CPK representation, close water molecules are shown transparently.

For the RGD peptide, bound states showed a strong preference for the cationic arginine residue as primary adsorption site; see Figure 5 and Figure 6. The most abundant bound conformation was C-N-N (22.26%), corresponding to single-point attachment via Arg (see Figure 5b and Table 3). The second most prevalent configuration, C-N-S (11.94%), features direct contact of Arg combined with solvent-separated interaction of the Asp residue. This pattern indicates that RGD frequently adopts an adsorption geometry in which the positively charged N-terminal arginine drives surface binding, while the negatively charged C-terminal aspartate remains separated from the surface by a hydration layer, likely reflecting electrostatic screening and hydration effects.

Taken together, these results demonstrate that peptide adsorption on the TiO_2_ surface occurs through well-defined, residue-specific conformations rather than random surface association. The high occurrence of the C-N-N-N state for KRSR and the C-N-N state for RGD underscores the dominant role of N-terminal cationic residues (lysine and arginine) in initiating and stabilizing direct surface contact. Cooperative and solvent-mediated binding configurations further highlight the dynamic nature of peptide–surface interactions and the importance of hydration effects in modulating adsorption geometry.

The presence of NaCl significantly affects not only the adsorption rate of the RGD and KRSR molecules but also greatly influences the configurations that emerge at the TiO_2_ interface. For KRSR, the non-adsorbed fraction increased from 18.34% to 48.99% in the presence of 200 mM NaCl, and the occurrence of the various configurations was markedly reduced (see Table 4). Many configurations that characteristically appear in the absence of NaCl (e.g., CCNC, NCNC, CNSC) could not be detected at all when NaCl was present. Although slight increases were observed in some cases (e.g., CCNN, NCNC, CSNN), the overall changes in microstate occurrence leads to a decrease in the overall adsorption on the surface.

A similar trend is observed for RGD, although the increase in the non-adsorbed fraction (from 21.93% to 36.68%) is considerably smaller in the presence of 200 mM NaCl (see Table 5). The decrease in the dominance of terminal binding is much more pronounced for RGD than for KRSR. In RGD, the rate of occurrence of states involving solvent-separated configurations increased in several cases (e.g., NNS, SNN, NSS). Although rearrangements among microstates are also detectable here, the reduction in the number and/or occurrence of microstates is far less dramatic, which is consistent with the macroscopically observed behavior.

## 4. Discussion

### 4.1. Summary of the Present Results

In this study, the adsorption behaviour of the bioactive peptides RGD and KRSR on an amorphous TiO_2_ surface was systematically investigated under a wide range of NaCl concentrations, spanning dilute to physiologically relevant ionic strengths. By combining multiple independent MD trajectories with quantitative descriptors of peptide stability, surface proximity, and adsorption dynamics, a statistically robust characterization was provided of how electrolyte conditions modulate peptide–surface interactions at the molecular level.

The backbone RMSD analysis demonstrates that neither RGD nor KRSR undergoes significant conformational destabilization upon adsorption, and peptide structural stability is largely insensitive to ionic strength. These findings rule out global conformational effects as the origin of the observed adsorption differences and indicate that salt-dependant behaviour is primarily governed by interfacial interactions rather than intrinsic peptide flexibility.

Clear peptide-specific trends emerge when considering surface proximity and adsorption persistence. For the positively charged KRSR peptide, increasing NaCl concentration leads to a statistically significant increase in the mean peptide-surface distance and a pronounced reduction in the fraction of time spent close contact with the TiO_2_ surface. In contrast, the adsorption behaviour of RGD remains remarkably robust across the same ionic strength range, with no significant dependence observed in either minimum distance or adsorption time ratio. KRSR carries a net charge of +3 at physiological pH, and its adsorption is therefore dominated by electrostatic attraction to negatively charged surface sites on hydrated TiO_2_ surfaces. Increasing ionic strength enhances electrostatic screening and introduces ion-mediated competition at the interface, weakening long-range Coulombic interactions and reducing adsorption stability. In contrast, RGD is overall charge-neutral but highly polar, enabling stable adsorption through hydrogen bonding and short-range polar interactions that are less sensitive to ionic screening effects.

The magnitude of the observed distance changes for KRSR, although modest on an absolute scale, translates into a substantial reduction in adsorption persistence. This highlights that even sub-ångström changes in average separation can have pronounced functional consequences for peptide residence time and interfacial stability.

### 4.2. Comparing with Previous Results

The present findings are in good agreement with earlier MD studies investigating peptide adsorption on crystalline TiO_2_ surfaces, while extending them in several important directions.

In our previous papers it was found that on anatase, rutile, and amorphous TiO_2_ surfaces, surface disorder enhances the heterogeneity of adsorption sites and broadens the distribution of peptide binding modes [21]. The current results build on this by demonstrating that, even on amorphous TiO_2_, peptide-specific electrostatic effects dominate the response to ionic strength, indicating that chemical functionality outweighs structural surface order in determining salt sensitivity. Specifically, for KRSR, earlier simulations on crystalline anatase (100) surfaces reported strong and persistent adsorption driven by lysine and arginine side chains [22]. The present study reveals that this strong binding is not universal but is significantly modulated by electrolyte concentration. This provides a possible explanation for discrepancies between in vitro adsorption experiments conducted under different buffer conditions and highlights the importance of explicitly accounting for ionic strength in both simulations and experimental design.

Beyond this, several additional computational works provide complementary context for interpreting the present results. Carravetta and Monti demonstrated with an early ab initio and classical MD study that adsorption configurations of short peptides at rutile TiO_2_ (110) are largely determined by a competition between direct peptide–surface electrostatics and the stability of the interfacial hydration layer, with charged termini and side chains (e.g., Lys/Arg) acting as primary anchoring motifs when they can partially displace surface-bound water [36]. This framework is directly consistent with the present observation that KRSR, dominated by cationic residues, shows adsorption that is readily weakened as electrolyte conditions change and screening/competition increases. In addition, in their other classical MD simulations they showed that model dipeptides on rutile TiO_2_ (110) reveals strong sequence dependence between acidic vs. basic residues, and emphasized that adsorption stability can be controlled by the balance of Coulomb attraction and solvent structuring at the interface [37]. This also supports that KRSR is an electrostatically driven binder whose residence time is sensitive to ionic conditions, whereas RGD can retain stable adsorption through short-range polar contacts that are less affected by screening.

For RGD specifically, multiple MD studies on rutile TiO_2_ (110) reported that adsorption involves the amino/guanidinium and carboxylate groups interacting with hydrophilic surface sites, while interfacial water substantially modulates binding geometry and kinetics [38,39,40]. Importantly, these works highlighted that adsorption dynamics depend strongly on how readily the peptide can maintain surface contacts in the presence of structured hydration layers, an interpretation consistent with the present finding that RGD adsorption is comparatively robust across a wide NaCl range. Moreover, the influence of the aqueous environment on RGD behaviour has been demonstrated explicitly by simulations comparing different water models, showing that subtle changes in hydration structure can shift peptide conformational dynamics and surface affinity. This supports the broader conclusion that RGD adsorption stability may be governed more by local hydrogen-bonding/hydration motifs than by long-range electrostatics, explaining its weak dependence on ionic strength in the present dataset.

Surface morphology also matters for dynamics: simulations comparing adsorption on perfect vs. nanotopographic rutile surfaces reported that nanoscale features can increase adsorption stability and alter adsorption pathways, partly by modifying local water structuring and available binding geometries [40]. This is relevant to the present amorphous-surface setting, where structural disorder introduces a broad distribution of adsorption microenvironments; however, our results indicate that, even under this heterogeneity, ionic strength still selectively destabilizes electrostatically dominated adsorption (KRSR) more than polar adsorption (RGD).

Kang et al. studied protein adsorption simulations comparing hydroxylated vs. non-hydroxylated rutile TiO_2_ (110) on a larger biomolecule scale and showed that surface hydroxylation state and hydration can dramatically alter adsorption propensity and interfacial energetics [41]. This reinforces the notion that electrolyte effects should be interpreted together with hydration-layer stability and surface chemistry, particularly when charged biomolecules are involved.

A recent work by Polimeni et al. examined the dynamics of a Ti-binding peptide at anatase (101) and emphasized that adsorption at TiO_2_ interfaces is highly dynamic and proceeds through metastable, water-mediated states rather than a single static bound pose [42]. This aligns with the approach taken in the present work (time fraction/residence analysis) and supports prioritizing adsorption persistence metrics when comparing sequences and solution conditions.

### 4.3. Implications and Limitations

From a biomaterials design perspective, the pronounced salt sensitivity of KRSR adsorption has important implications for its application in implant surface functionalization. While KRSR is known to promote selective osteoblast adhesion, the present results suggest that its interfacial stability may vary significantly with local electrolyte conditions. This may contribute to variability in experimental outcomes reported under different buffer compositions and highlights the importance of considering physiological ionic strength when evaluating peptide coatings. In contrast, the adsorption behaviour of RGD across a broad NaCl concentration range supports its continued use as a reliable bioactive motif under physiologically relevant conditions. The differing response of RGD and KRSR further suggests that combining peptides with complementary interaction mechanisms, or designing hybrid sequences that balance electrostatic and polar contributions, may improve the stability and predictability of biofunctionalized Ti-based implants [6,7,43].

Despite these insights, several limitations of the present study should be acknowledged. First, the simulations were performed using classical force fields, which do not explicitly account for electronic polarization or charge transfer effects at the interface. While the employed parametrization has been validated against adsorption properties, polarization effects may become relevant for highly charged residues and could influence absolute interaction strengths.

The simulations were performed using NaCl as the sole electrolyte to isolate the effect of ionic strength on electrostatic screening at the peptide-TiO_2_ interface. This choice enables systematic comparison of adsorption behaviour under controlled electrolyte conditions, without introducing additional ion-specific adsorption or surface-bridging mechanisms. In physiological environments, divalent cations such as Ca^2+^ and Mg^2+^ are present at millimolar concentrations and can introduce different interaction pathways, including ion-mediated surface bridging, coordination to surface oxygen atoms, and stabilization of negatively charged peptide residues. These effects go beyond simple electrostatic screening and define a distinct physiochemical regime involving competitive adsorption and localized binding. Extending the present approach to multivalent ions represents an important direction for future work.

All simulations in the present study were performed at a near fixed temperature of 310 K, corresponding to physiological conditions. While temperature can influence peptide conformational dynamics, interfacial hydration structure, and adsorption kinetics, it was not treated as a variable parameter here. The reported trends reflect the effect of ionic strength on peptide adsorption at constant physiological temperature. Exploring temperature-dependent adsorption behaviour, including potential changes in hydration-mediated interactions and kinetic stability, represents an interesting direction for future work but lies beyond the scope of the present study.

The peptides investigated here are short motifs studied in isolations. In vivo, these sequences are often presented as part of larger proteins or immobilized through surface linkers, which may restrict conformational freedom and alter adsorption dynamics. While the present results capture intrinsic peptide–surface interactions, extrapolation to complex biological context should be made with caution.

Within these limitations, the present study provides a mechanistically grounded and quantitatively robust assessment of electrolyte effects on peptide adsorption to amorphous TiO_2_ surfaces and contributes to bridging the gap between idealized simulation models and physiologically relevant implant environments.

## 5. Conclusions

In this work, MD simulations were used to investigate the adsorption of RGD and KRSR peptides on an amorphous TiO_2_ surface over a wide range of NaCl concentrations. Both peptides remained structurally stable upon adsorption, indicating that ionic strength does not induce significant conformational changes. However, a pronounced peptide-specific response to electrolyte concentration was observed. Adsorption of the positively charged KRSR peptide was progressively weakened with increasing NaCl concentration, as reflected by increased peptide–surface separation and reduced surface residence time, consistent with electrostatic screening effects. In contrast, the adsorption behaviour of RGD was largely insensitive to ionic strength, suggesting stabilization by short-range polar interactions. These results highlight the importance of accounting for physiological electrolyte conditions when modelling and designing peptide-functionalized Ti-based implant surfaces.

## Figures and Tables

**Table 1:** Composition of the simulation systems at different NaCl concentrations. For each ionic condition, the number of Na^+^ and Cl^−^ ions and the corresponding number of water molecules are listed. All systems were charge-neutral and solvated using the TIP3P water model.

NaCl Concentration (mM)	Na^+^ Ions	Cl^−^ Ions	Water Molecules
1	1	1	13,209
25	6	6	13,193
50	12	12	13,185
75	18	18	13,171
100	24	24	13,161
125	30	30	13,148
150	37	37	13,134
175	41	41	13,123
200	47	47	13,115

System charge neutrality was maintained by adding equal numbers of Na^+^ and Cl^−^ ions for RGD and appropriate counterions for the charged KRSR peptide (3 more Cl^−^ ions since the KRSR has 3+ charges).

**Table 2:** Observed combinatorial conformations of the KRSR peptide on the TiO_2_ surface, ranked by decreasing occurrence. C indicates direct peptide surface contact (~0.2 nm), S a solvent-separated (water-mediated) state (~0.4 nm), and N a non-adsorbed configuration (>0.5 nm). Only conformations with an occurrence rate exceeding 1% are included.

Residue	Distance															
K (Lys)	N	C	N	N	C	C	N	N	C	N	S	S	N	C	C	N
R (Arg)	N	N	N	C	N	C	C	S	N	N	S	N	S	N	S	C
S (Ser)	N	N	N	N	N	N	N	N	N	N	N	N	N	S	N	N
R (Arg)	N	N	C	N	C	N	C	C	S	S	C	N	N	N	N	S
Rates (%)	42.6	10.5	6.2	5.4	4.4	3.8	2.8	1.9	1.9	1.8	1.8	1.5	1.4	1.4	1.3	1

C ≈ 0.2 nm (contact); S ≈ 0.4 nm (solvent-separated); N > 0.5 nm (non-adsorbed). Values indicate time fractions of the simulations in the given state.

**Table 3:** Observed combinatorial conformations of the RGD peptide on the TiO_2_ surface, ranked by decreasing occurrence. C indicates direct peptide surface contact (~0.2 nm), S a solvent-separated (water-mediated) state (~0.4 nm), and N a non-adsorbed configuration (>0.5 nm). Only conformations with an occurrence rate exceeding 1% are included.

Residue	Distance											
R (Arg)	N	C	C	N	C	S	C	N	C	N	S	N
G (Gly)	N	N	N	N	S	N	S	S	N	S	N	N
D (Asp)	N	N	S	S	N	N	S	N	c	S	S	C
Rates (%)	34.1	22.3	11.9	9.8	4.4	3.1	2.7	2.7	2	1.8	1.5	1.1

C ≈ 0.2 nm (contact); S ≈ 0.4 nm (solvent-separated); N > 0.5 nm (non-adsorbed). Values indicate time fractions of the simulations in the given state.

**Table 4:** Observed combinatorial conformations of the KRSR peptide on the TiO_2_ surface at 1 mM NaCl and at 200 mM NaCl, ranked by decreasing occurrence. C indicates direct peptide surface contact (~0.2 nm), S a solvent-separated (water-mediated) state (~0.4 nm), and N a non-adsorbed configuration (>0.5 nm). Only conformations with an occurrence rate exceeding 2% are included.

Residue	Distance											
K (Lys)	N	C	N	C	N	C	N	C	N	N	C	C
R (Arg)	N	N	N	N	S	C	C	C	S	C	S	N
S (Ser)	N	N	N	N	N	N	N	N	N	N	N	S
R (Arg)	N	N	C	C	C	N	N	C	N	C	N	C
Rates (% at 1 mM)	18.34	13.56	12.99	12.70	5.36	4.62	4.03	2.70	2.55	2.45	2.40	2.25
Rates (% at 200 mM)	48.99	8.49	5.00	0.14	3.31	7.07	8.22	0.00	2.52	0.00	2.58	0.00

C ≈ 0.2 nm (contact); S ≈ 0.4 nm (solvent-separated); N > 0.5 nm (non-adsorbed). Values indicate time fractions of the simulations in the given state.

**Table 5:** Observed combinatorial conformations of the RGD peptide on the TiO_2_ surface at 1 mM NaCl and at 200 mM NaCl, ranked by decreasing occurrence. C indicates direct peptide surface contact (~0.2 nm), S a solvent-separated (water-mediated) state (~0.4 nm), and N a non-adsorbed configuration (>0.5 nm). Only conformations with an occurrence rate exceeding 1% are included.

Residue	Distance								
R	C	N	C	C	N	C	S	N	N
G	N	N	N	S	N	S	N	S	S
D	N	N	S	S	S	N	N	N	S
Rates (% at 1 mM)	34.62	21.93	15.06	8.16	6.62	5.33	2.42	2.07	1.67
Rates (% at 200 mM)	19.60	36.68	13.52	5.87	12.22	3.19	2.73	2.04	1.77

C ≈ 0.2 nm (contact); S ≈ 0.4 nm (solvent-separated); N > 0.5 nm (non-adsorbed). Values indicate time fractions of the simulations in the given state.

## Data Availability

The original raw data contributions presented in this study are included in the article/Supplementary Materials. Further inquiries can be directed to the corresponding author.

## Supplementary Materials

The following supporting information can be downloaded at: https://www.mdpi.com/article/10.3390/biom16020336/s1. The supplementary materials include the final GROMACS run input files for MD simulations of RGD and KRSR peptides on TiO_2_ surfaces at different NaCl concentrations (one tpr file at every NaCl concentration), together with the corresponding raw analysis outputs of the RMSD (gmx rms) minimal distances (gmx mindist), raw files including the initial coordinate files, topology and scripts for the production and analysis.

## Abbreviations

The following abbreviations are used in this manuscript:

MD	Molecular dynamics
RMSD	Root-mean-square deviation
PME	Particle mesh Ewald
PBC	Periodic boundary conditions
RGD	Arginine–glycine–aspartic acid (peptide motif)
KRSR	Lysine–arginine–serine–arginine (peptide motif)
TiO_2_	Titanium dioxide
NaCl	Sodium chloride
CHARMM	Chemistry at HARvard Macromolecular Mechanics
TIP3P	Transferable intermolecular potential, 3-point (water model)
NVT	Constant number of particles, volume, and temperature
NPT	Constant number of particles, pressure, and temperature
V-rescale	Velocity-rescale thermostat
